# 46, XX disorder of sexual development associated with mixed germ cell tumor of the prostate: a rare case report

**DOI:** 10.1186/s12894-024-01420-z

**Published:** 2024-02-12

**Authors:** Changrong Wang, Jiangli Du, Xueping Xiang, Yuyong Wang, Jingjing Xiang, Qiaoping Xu

**Affiliations:** 1 Department of Pathology, Affiliated Hangzhou First People’s Hospital, Westlake University School of Medicine, Hangzhou, 310006 China; 2 Hangzhou Buping Medical LaboratoryCo., Ltd, Hangzhou, 310006 China; 3 Department of Pathology, Affiliated Second Hospital, Zhejiang University School of Medicine, Hangzhou, 310006 China; 4 Department of Urology, Affiliated Hangzhou First People’s Hospital, Westlake University School of Medicine, Hangzhou, 310006 China; 5 Department of Clinical Pharmacology, Affiliated Hangzhou First People’s Hospital, Westlake University School of Medicine, Hangzhou, 310006 China

**Keywords:** 46 XX, Disorder of sex development(DSD), Germ cell tumour, Prostate

## Abstract

**Background:**

Extragonadal germ cell tumors originating from the prostate are exceptionally rare. To the best of our knowledge, there have been no reported cases of mixed germ cell tumors in individuals with 46 XX disorder of sex development. In this study, we conducted a comprehensive analysis using whole genome sequencing to investigate the clinicopathological and molecular genetic characteristics of a submitted case, with the objective of elucidating its underlying pathogenesis.

**Case presentation:**

A 40-year-old male patient was diagnosed with a combination of 46, XX disorder of sex development and a primary prostate mixed germ cell tumor with yolk sac tumor and teratoma components. Whole-genome sequencing revealed that the tumor cells had a high somatic mutational load. Analysis of genomic structural variations and copy number variants confirmed the patient's karyotype as 46, XX (SRY +). Additionally, the patient exhibited short stature, small bilateral testes, slightly enlarged breasts, elevated serum alpha-fetoprotein concentrations, elevated follicle-stimulating hormone and luteinizing hormone levels, and low testosterone levels.

**Discussion:**

A case of 46, XX disorder of sex development, along with a primary prostatic mixed germ cell tumor, was diagnosed. This diagnosis has contributed to advancing our understanding of the genetic and phenotypic profile of the disease and may provide some insights for its treatment.

## Background

Disorder of sex development (DSD) is a group of diverse conditions that arise from abnormalities in the sexual differentiation process [1]. These abnormalities can result in differences between chromosomal sex, gonadal sex, and/or phenotypic sex. The disease is classified into three main categories: 46, XY disorders of sex development (46, XY DSD), 46, XX disorders of sex development (46, XX DSD), and DSD with sex chromosome abnormalities [1]. The pathogenesis of the disease is complex and involves genetic variations in human sex determination genes. In addition to well-known genes like SRY, SOX9, and NR5A1, recent research has shown that alternative regulatory pathways, such as DHH, MAPK signaling pathway, and ubiquitination, can also contribute to the development of the disease. Mutations in the SRY gene account for approximately 10–15% of individuals with DSD [2], while genes like SOX9, DHH, NR5A1, and MAP3K1 are mutated in around 30% of cases. Currently, the genetic cause is identified in less than 50% of cases.

46, XX DSD primarily presents as external genitalia abnormalities during childhood and can lead to infertility, azoospermia, gynecomastia, and sexual dysfunction in adulthood. In the majority of cases, individuals with 46, XX DSD are caused by a translocation of a segment of the Y chromosome that carries the SRY gene during meiosis [3]. The presence of the SRY gene may increase the risk of developing gonadal tumors, specifically gonadal germ cell tumors [4] or testicular Leydig tumors [5]. However, there have been no reported cases of extragonadal germ cell tumors associated with this disease.

In this academic report, we present a case of 46, XX testicular DSD with a primary prostatic mixed germ cell tumor, which is an extremely rare occurrence. We delve into the clinicopathological features and genetic characteristics of the case to gain a better understanding of the disease's pathogenesis.

The study received approval from the Research Ethics Committee of Hangzhou First People's Hospital (Ethics approval number: IIT-20230630–0142-01). Informed consent was obtained from the patient's wife.

## Case presentation

**Fig. 1:**
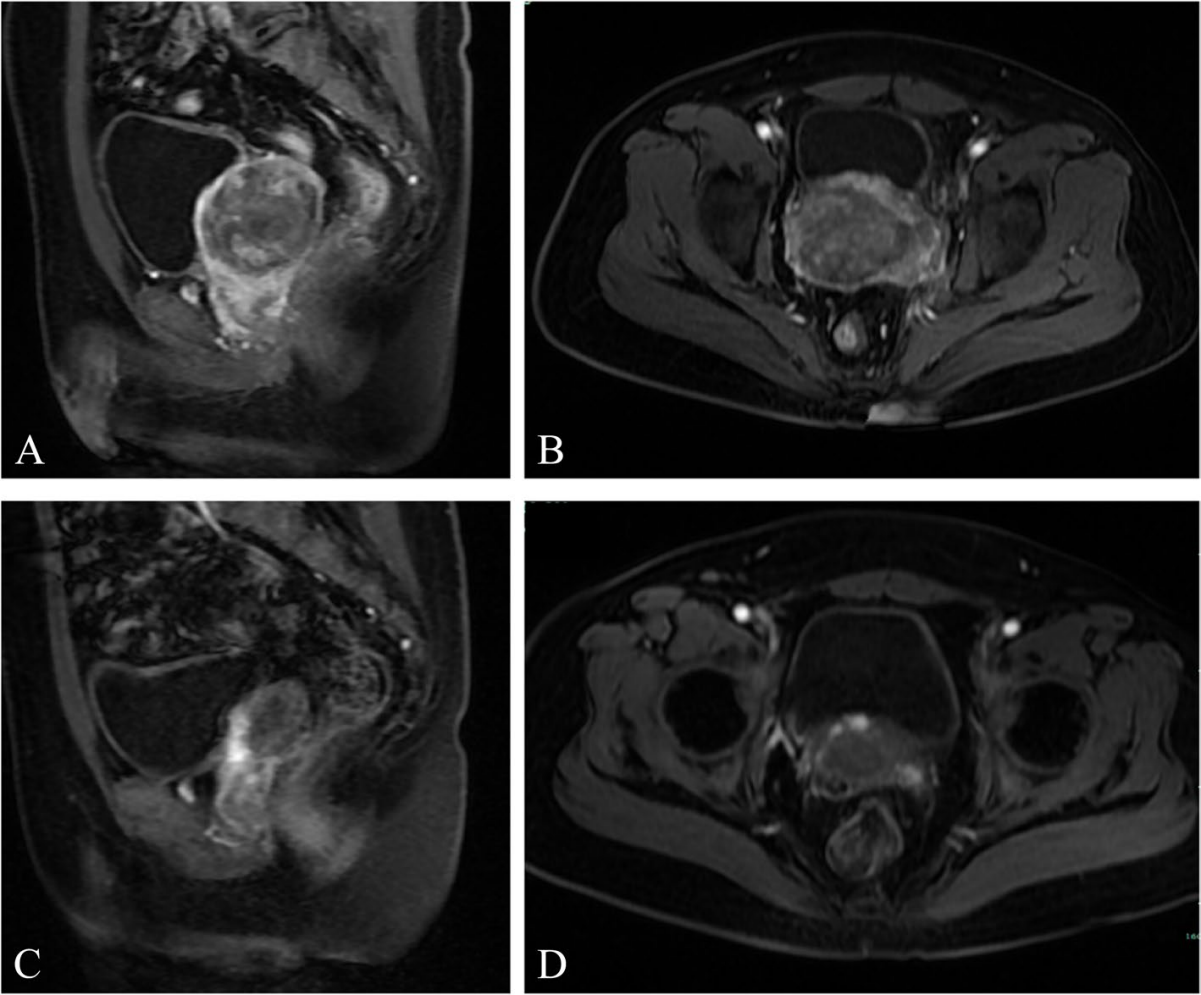
Prostate MRI images **A** In the pre-chemotherapy images, the sagittal T2-weighted images (T2WI) indicated the presence of a mass in the middle and upper regions of the prostate. The mass showd mixed signals and had unclear boundaries. **B** In the T1-weighted imaging (T1WI) sequence, fat inhibition revealed clear and uneven enhancement surrounding the tumor, without any internal enhancement. **C**, **D** After chemotherapy treatment, the size of the mass and the lymph nodes in the left iliac vessel noticeably decreased

**Fig. 2:**
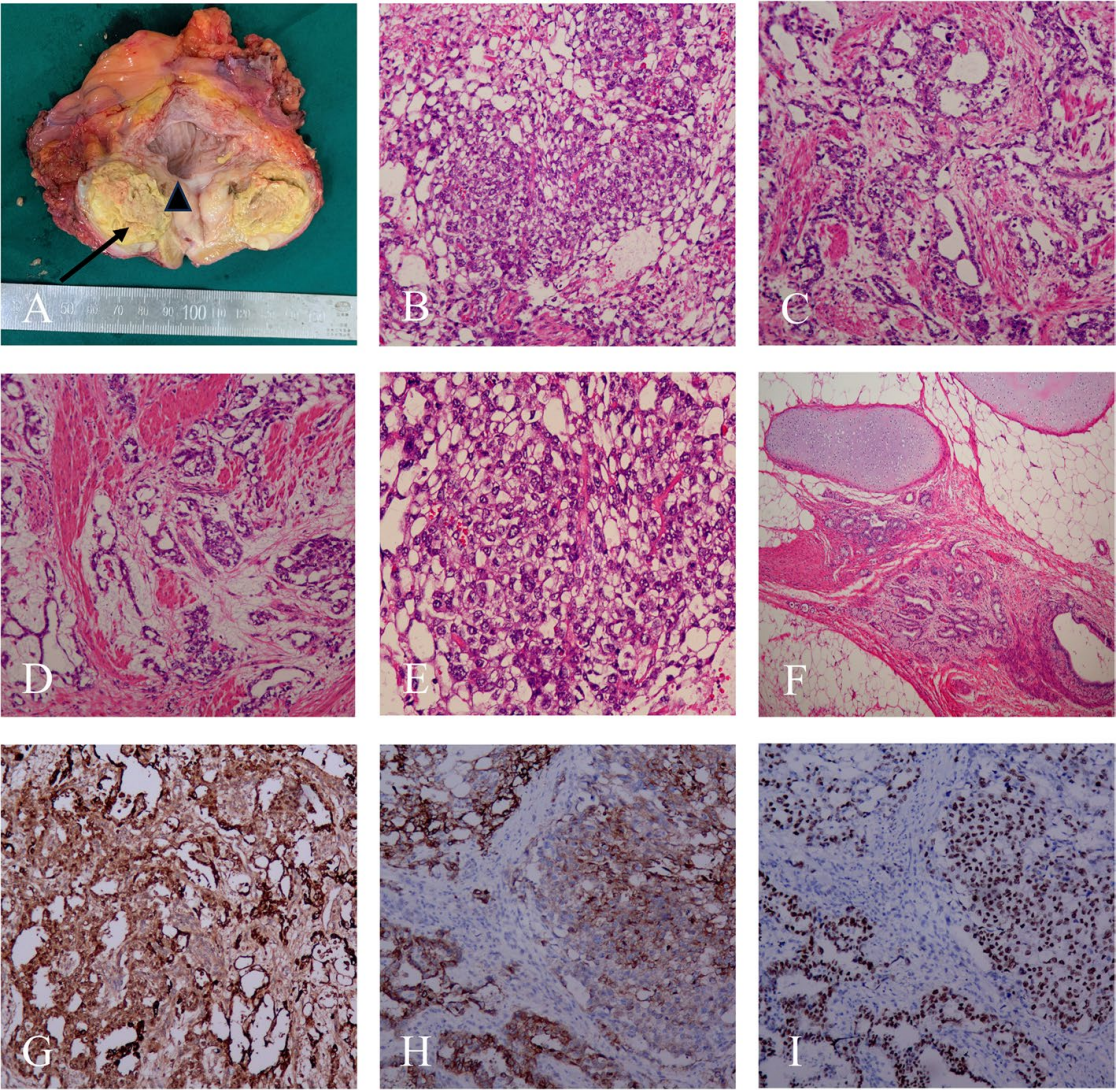
**A** Macroscopic examination of radical cystectomy of the prostate. The tumor invaded the entire prostate parenchyma, displaying visible necrosis, yellow color and soft texture (the arrow). The tumor partially infiltrated the posterior bladder wall(the triangle). Microscopic examination revealed YST components(B-E), including microcystic **B** small tubular **C** and myxoid types **D** H&E 100 × **E** YST tumor cells were large with nuclear atypia and rich eosinophilic or transparent cytoplasm, H&E 200 × . **F** Teratoma components included cartilage, mucinous glands, and ciliated columnar epithelial glands. H&E 100 × . Immunohistochemical examination of YST revealed positive results for AFP **G** Glypican3 **H** and SALL4 **I** 100 ×

A 40-year-old male patient presented with progressive dysuria and pain without any obvious cause. The patient had a history of cardiac disease and had undergone coronary stent placement two months earlier following a myocardial infarction. In terms of the patient's family history, The maternal grandmother of the patient's father and mother were sisters, they were within three generations of consanguineous marriage. Physical examination, the patient's height was measured at 163 cm and weight at 63.5 kg. The patient had fair skin, sparse beard, and sparse armpit hair. The breasts were slightly enlarged and the penis showed dysplasia, while the foreskin was normal and there was no entrapment. The testicles were dysplastic, measuring about 2 cm x 1 cm × 0.5 cm and were in a symmetrical position with no abnormal nodules detected. The patient's laboratory results revealed an elevated AFP value of 17,066.16 μg/L (reference range 0.25). The testosterone level was within the normal range at 3.6 nmol/L (reference range 3.0 ~ 28.2). However, the levels of Follicle-Stimulating Hormone (FSH) and Luteinizing Hormone (LH) were elevated at 46.3 IU/L (reference range 1.4 ~ 18.0 IU/L) and 36 IU/L (reference range 1.5 ~ 9.3 IU/L), respectively. The magnetic resonance imaging (MRI) of the prostate revealed the presence of a noticeable mass (measuring 8 cm) in the middle and upper regions. The mass exhibited internal hemorrhage and necrosis, and it had extended to the bilateral seminal vesicles, the posterior wall of the bladder, and the surrounding adipose space. Additionally, multiple lymph node metastases were observed in close proximity to the left iliac vessel (Fig. 1A, B). The patient underwent a prostate biopsy guided by ultrasound, which revealed a yolk sac tumor (YST). A CT scan of the abdomen ruled out the possibility of a testicular tumor. The patient was diagnosed with an extragonadal germ cell tumor with multiple metastases and was scheduled to receive neoadjuvant therapy as first-line treatment. Six cycles of chemotherapy using etoposide and cisplatin were administered. Subsequent MRI revealed a reduction in the size of the mass to 3 cm and a decrease in enhancement (Fig. 1C, D). Following this, radical cystectomy and prostatectomy were performed. Gross examination, the tumor had completely invaded the prostate. The majority of the mass was necrotic, yellow in color, and had a soft texture (Fig. 2A). Microscopic examination, the remaining tumor primarily consisted of YST, accounting for approximately 90% of the tumor, while a smaller portion (approximately 10%) was identified as teratoma. The YST exhibited various histological patterns, including microcystic, tubular, and mucinous. The teratoma components consisted of cartilages, mucoepithelial glands, and ciliated columnar epithelial glands (Fig. 2B-F). Immunohistochemistry, YST tumor cells were positive for AFP, GPC-3, SALL4, CD117 and PLAP, and negative for D2-40, CD30, EMA, HCG and OCT3/4. The proliferation index of Ki-67 was 80% (Fig. 2G-I). The tumor had invaded the bilateral seminal vesicles. One lymph node of the left external iliac area showed YST metastasis.

Following the surgical procedure, the patient underwent 18 sessions of radiotherapy. However, the treatment had to be discontinued due to the patient's pronounced weakness, abdominal distension, and severe nausea. Unfortunately, the patient succumbed to various complications five months after the surgery, including the spread of tumors in the abdominal cavity, acute renal failure, cardiac failure, ventricular aneurysm, severe myelosuppression, hepatic insufficiency, hyponatremia, hyperuricemia, and fungal infection.

**Fig. 3:**
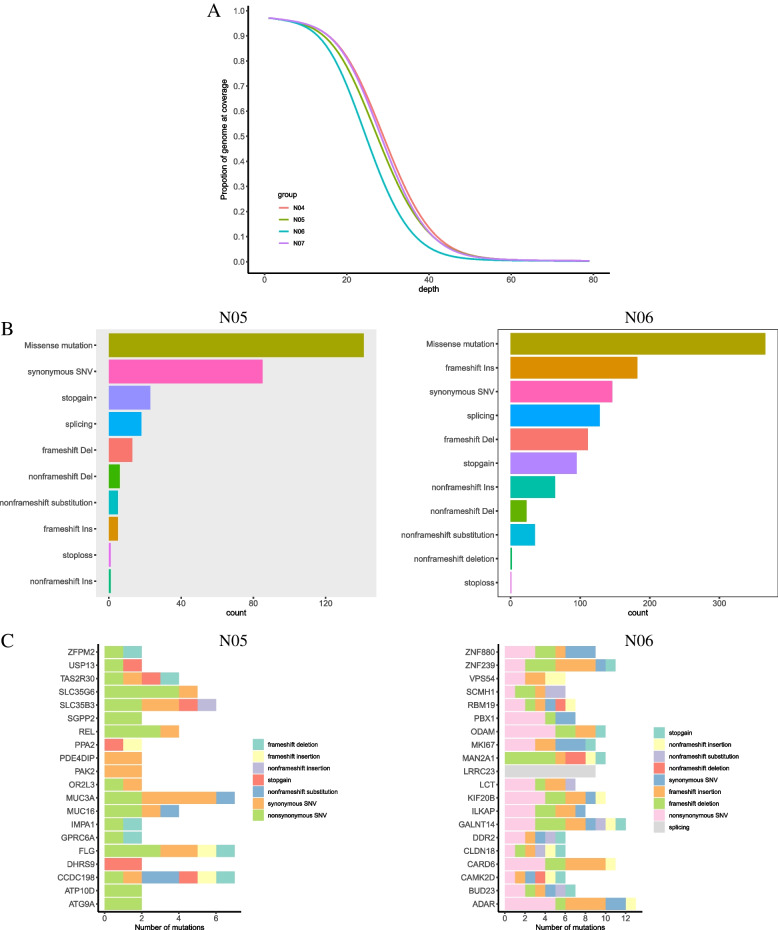
Whole-genome sequencing of mutations explored both the tumor and the surrounding normal tissue. **A** Sequencing depth and coverage of the samples. NO4 referes to the surrounding normal prostate. NO5 referes to the YST component and NO6 referes to the teratoma component. **B** Distribution of different somatic mutation types across the genome. **C** Top 20 genes containing the highest number of mutations in the exon/intron region

We performed whole-genome sequencing (WGS) on the patient's prostate tumor and surrounding normal prostate tissue from the patien. The samples were sequenced at about 30X (Fig. 3A). The YST component had 444 mutations in 365 genes, while the teratoma component had 1701 mutations in 819 genes, as compared to the normal prostate tissue. Missense mutations were the most prevalent variants in both YST and teratoma, although YST also had a significant number of synonymous SNVs. Furthermore, the teratoma exhibited rameshift ins, synonymous SNVs, splicing, frameshift del, and stopgain variants. (Fig. 3B). Among the most frequently mutated genes in the exon region, genes in YST exhibited nonsynonymous SNV changes, while teratoma exhibited both nonsynonymous SNVs and frameshift insertions/deletions (Fig. 3C). However, classical pathogenic genes, such as SRY and SOX9, as well as other related pathogenic genes, like NR5A1, DHH, and MAPK1, did not display any small mutations.

**Fig. 4:**
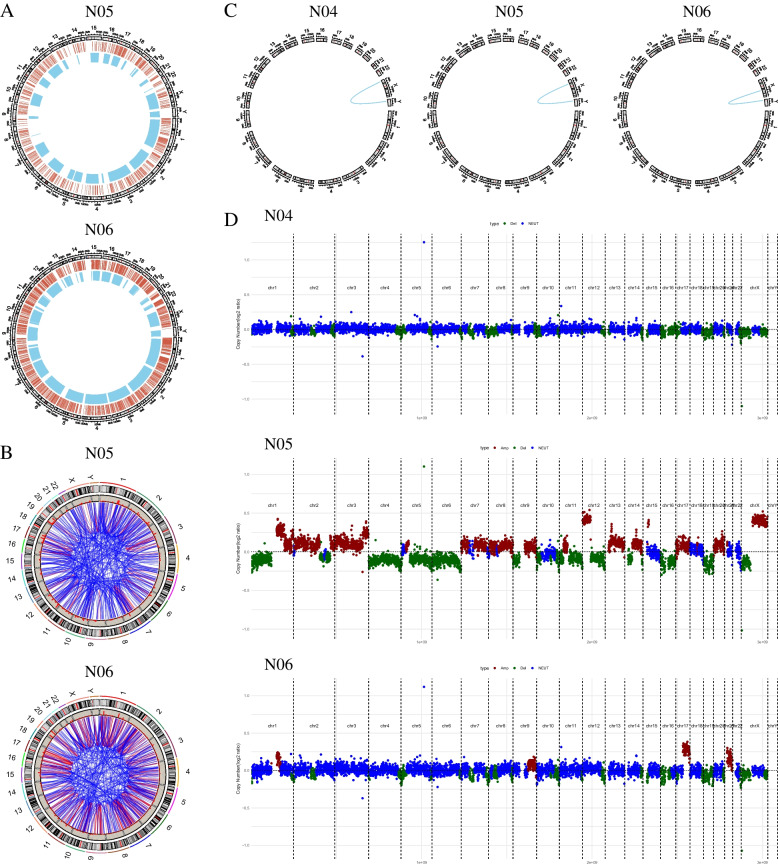
**A** Circos plots of the YST and teratoma tumour tissue showed the number of small mutations (red) and structural variants (blue). **B** Circos plots of the YST and teratoma tumour tissue revealed a high number of intrachromosomal (red) and interchromosomal (blue) translocations. **C** Circos plots of of the YST, teratoma and normal prostate tissue showed the interchromosomal translocation of the SRY gene. **D** Copy-number variations (CNVs) profiles of whole genome sequencing in the YST, teratoma and normal prostate tissue. One dot on the plot refers to the read-depth ratio summarized by one probe; red indicates gene gain, green gene loss and blue gene normal

A significant proportion of genes with insertions were observed among the top 20 mutated genes. This observation led us to further analyze insertions and structural variations (SVs) to understand the potential origins of these alterations through major chromosomal events. Our analysis revealed a high number of genomic SVs in both tumor components, with 426 SVs in the YST and 599 SVs in the teratoma (Fig. 4A). These SVs encompassed deletions, duplications, inversions, and translocations. Notably, chromosomal translocations, both intrachromosomal and interchromosomal, were the most frequent structural events observed. (Fig. 4B). Additionally, we detected the key pathogenic chrX and chrY interchromosomal translocation in both tumor and normal prostate tissue, that was, the translocated SRY gene appeared at the end of the short arm of the chrX (Fig. 4C). This finding is consistent with the karyotype 46, X, der(X) t(X;Y)(p22.3;p11.2) identified in our previous detectation [6]. The SRY gene is located in chrY 2786855–2,787,682, which encodes a protein that is a testicular determinant and initiates male sex determination.

Since copy number variations (CNVs) is an essential part of genomic structural variation and a common etiology of many congenital abnormalities such as early abortion, developmental delay, intellectual disability, autism, and congenital heart disease, we additionally conducted CNVs analysis on both tumor and normal prostate tissue samples. The results showed substantia genomic copy number amplification on chr12 and chrX (Fig. 4D). Moreover, the patient's karyotype of 46, XX was supported by CNVs analysis.

## Discussion

46, XX DSD is a rare disorder of sex development that can be classified as SRY positive and SRY negative. SRY positive cases account for approximately 90%, while SRY negative cases account for around 10% [7]. In 46, XX (SRY +) males, the condition often results from de novo X–Y chromosomal translocations. These translocations happen when the father's X and Y chromosomes exchange during meiosis, leading to the translocation of the SRY gene to the end of the short arm of the X chromosome [8, 9]. However, translocation to the end of the long arm of the X chromosome [10] and autosomes [11] have also been reported. The karyotype of the parent's chromosomes is typically normal, but in certain circumstances, it can be passed down from the father carrier to the next generation [12]. Xing Ya et al. [13] reported six cases of 46, XX males. Only one case was found to have an abnormal band at the end of the short arm of the X chromosome, while the remaining five cases were normal. This indicates that most of the 46, XX (SRY +) males belonged to an occult translocation. Our previous study revealed that the patient identified as 46, XX (SRY +) male through karyotyping and fluorescence in situ hybridization [6].

The clinical presentation of 46, XX DSD varies depending on the presence of the SRY gene. Most individuals with 46, XX (SRY +) males do not show deformities in their reproductive organs and are typically diagnosed in adulthood due to symptoms such as short testicles, infertility, or enlarged breasts. These individuals have lower height compared to normal males, with an average height of (166 ± 6.5) cm. This is likely due to insufficient androgen secretion and the deletion of growth control gene on the long arm of the Y chromosome. The patient's height was 163 cm, which was lower than the average height. This observation is consistent with the characteristics of the disease. In addition, patients with this disease often exhibit low testosterone levels and testicular dysplasia, resulting in underdeveloped secondary sexual characteristics such as sparse pubic hair, armpit hair, and a less prominent Adam's apple The physical examination of the patient confirms these characteristics. The patient's serum testosterone levels were within the range of normal low values, and both FSH and LH were elevated, which is also consistent with the hormonal changes of the disease.

Previous research on 46, XX DSD has primarily focused on the condition itself. However, it has also been observed that individuals with DSD have a higher risk of developing germ cell tumors [14]. Studies have found a strong correlation between the presence of Y chromosome segments and the location of the gonads with the occurrence of these tumors [15]. Neoplastic transformation can be observed in up to 30% of cases of hereditary gonadal dysfunction when Y chromosome fragments are detected. However, it is important to note that 46,XX patients without Y chromosome-associated fragments do not have a high risk of developing tumors. Carcavilla et al. [4] reported a case of 46, XX(SRY +) male child with undifferentiated spermatogenic tumor of unknown genitalia and spermatogenic tubule, while Osaka et al. [5] documented a rare case of 46,XX(SRY +) male patient with testicular Leydig cell tumor. The occurrence of this scenario can be attributed to two factors. Firstly, the SRY gene is expressed in adult Sertoli cells and germ cells, and it plays a crucial role in the development and maintenance of germ cell function. Any alteration in the SRY gene can lead to gonadal dysplasia. Secondly, dysplastic gonadal tissue tends to have more immature structures, which increases the risk of tumor development [16]. The patient presented with a primary prostate mixed germ cell tumor composed of YST and teratoma. To the best of our knowledge, this specific combination has not been previously documented in the existing literature.

Male germ cell tumors primarily arise in the testis, with seminoma being the most prevalent subtype. Conversely, extra-gonadal germ cell tumors typically occur in midline structures and organs, with the mediastinum and central nervous system being the most common sites. Among extra-gonadal germ cell tumors, teratoma is the most frequently observed type. When an extragonadal germ cell tumor is detected, it is crucial to determine its origin from the testis. However, conducting a histological examination by excising both testes is often impractical in clinical practice. If the physical examination and imaging studies do not reveal any abnormalities, there is no need for testicular biopsy or removal. In this case, the patient initially declined bilateral testicular biopsy. However, the physical examination and imaging studies did not indicate any signs of a suspicious testicular tumor, although the possibility of an early testicular germ cell tumor could not be completely ruled out.

The etiology of extra-gonadal germ cell tumors is still not fully understood, and two main theories have been proposed. The first theory suggests that during embryonic development, primordial germ cells associated with yolk sac endodermal differentiation migrate to the gonad through the urogenital ridge. Some of these cells may remain undifferentiated. The second theory proposes that pluripotent stem cells, which transform into germ cells during the blastocyst stage of embryonic development, may migrate to other organs and persist there. During growth and development, most primitive cells degenerate, but a few non-degenerate cells retain their differentiation potential. These cells, when stimulated by certain factors, can potentially transform into germ cell tumors. In embryonic development, primordial germ cells migrate to different regions such as the prostate, thymus, retroperitoneal organs, and pineal gland. The migration to these regions is explained by the former mechanism, while the migration to the liver, stomach, and lung is explained by the latter mechanism [17–19].

Based on Whole Genome Sequencing (WGS) results of the patient's tumor tissue, no previously reported disease-related mutations were detected within the genes. However, numerical mutations and structural variations (SVs), including genomic deletions, duplications, inversions, and translocations, were observed. The patient's tumor exhibits characteristics akin to type I Germ Cell Tumors (GCTs) that occur in extragonadal tissues. The histopathological progression from teratoma to Yolk Sac Tumor (YST) supports this diagnosis. Furthermore, the overall near-diploid nature of the entire tumor reinforces this categorization. Notably, the YST component displays a higher frequency of numerical mutations compared to the teratoma component, which is consistent with the development of YST through the transformation of teratoma components. Type I GCTs typically manifest in neonates and are rare beyond six years of age but can also present in post-pubertal males affecting both testicular and extragonadal locations, such as the mediastinum and brain, or even near the prostate as seen in this case report. These tumors potentially arise from early migratory primordial germ cells reprogrammed into embryonic stem cell-like states, capable of multilineage differentiation. The extensive history of the tumor – presenting at 40 years of age with origins dating back to embryonic life – could account for the multitude of mutations, SNVs, and other genomic abnormalities. Despite these findings, it remains challenging to definitively exclude the possibility that the patient's tumor might be a type II GCT. This uncertainty arises because there was no pathological evidence of an in situ germ cell tumor in the patient's testes, coupled with their presentation of underdeveloped gonads characterized by a typical 46, XX Disorder of Sex Development (DSD). It is noteworthy that the presence of the Y chromosome significantly increases the risk for developing gonadal and extragonadal type II GCTs. Given the complex interplay between the patient's clinical profile, genetic alterations, and pathological features, pinpointing the exact tumor classification is difficult. It may be more apt to classify it according to Oosterhuis JW's proposed type VI category, which shares similarities with either type I or the non-seminoma components of type II GCTs, but lacks seminomatous elements [20].

## Conclusion

In summary, the occurrence of a primary prostatic mixed germ cell tumor composed of YST and teratoma in a patient with 46 XX DSD is an exceptionally rare phenomenon in clinical practice. This case study has expands the clinical phenotypic spectrum of 46, XX DSD and provides a valuable reference for understanding the mechanisms underlying the occurrence and development of gonadal germ cell tumors.

## Methods

### Whole-genome sequencing

The genomic DNA from the formalin-fixed paraffin-embedded tissues of the patient's prostate tumor, lymph node metastasis tumor, and normal prostate were extracted using a DNA extraction kit (Hieff NGS® OnePot Pro PCR-Free DNA Library Prep Kit for Illumina, 12209ES96), following the instructions provided in the kit. The gDNA samples were were normalized to the concentration and volume required for the PCR-free library preparation kit using the Illumina Novaseq platform-specific TruSeq library high-throughput library preparation kit for sequencing libraries. The fragments were randomly broken into about 300 bp fragments using a Covaris breaker. After end repair and A-tail addition, the DNA libraries were prepared by ligating connectors at both ends of the fragments. The insert size of each library was assessed using the Agilent 2100 kit, ensuring that the average fragment size was between 220 and 350 bp. The concentration of each library was calculated using the KAPA KK4824 NGS Library Quantification kit to ensure that the concentration of each library was > 10 ng/ul. Sequencing was performed on an Illumina Novaseq 6000 high-throughput sequencing platform.

### Bioinformatic analysis

Data with tissue sample coverage of 30X were transmitted in the form of raw sequencing reads (compressed FASTQ file format). The human genome assembly GRCh38(hg38) was used as a reference gene, and BWA men (version 0.7.17) was used for comparison. Sort the output SAM file and convert it to BAM format. Base recalibration was performed using Genome Analysis Toolkit (GATK version 4.2.5.0). Somatic point mutations (SNPs) were identified and analyzed using GATK's HaplotypeCaller module (GATK version 4.2.5.0). Structural variations (SVs) were analyzed using delly software (version v0.9.1). The R package RCircos (version 1.2.2) was used to visualize structural variations, while ichorCNA analysis was used to visualize copy number variations (CNVs).

## Data Availability

The datasets generated and/or analysed during the current study are available in the Sequence Read Archive of NCBI repository, SRA accessions: SRR25620886, SRR25620887, SRR25620885, SRR25620884.( https://www.ncbi.nlm.nih.gov/sra/).

## Abbreviations

DSD: Disorder of sex development
YST: Yolk sac tumor
FSH: Follicle-Stimulating Hormone
LH: Luteinizing Hormone
CNVs: Copy-number variations
SVs: Structural variations
GCNIS: Germ cell neoplasm in situ

## References

[1] Yamada G, Suzuki K, Haraguchi R, Miyagawa S, Satoh Y, Kamimura M, Nakagata N, Kataoka H, Kuroiwa A, Chen Y (2006). Molecular genetic cascades for external genitalia formation: an emerging organogenesis program. Dev dynam.

[2] Hughes IA (2008). Disorders of sex development: a new definition and classification. Best Pract Res Clin Endocrinol Metab.

[3] Rajender S (2006). SRY-negative 46, XX male with normal genitals, complete masculinization and infertility. Mol Hum Reprod.

[4] Carcavilla A, Alonso M, Ezquieta B, García-Galloway E, Barrio R, Nistal M (2008). An XX male with an intratubular undifferentiated germ cell neoplasia. Fertil Steril.

[5] Osaka A, Ide H, Matsuoka K, Iwahata T, Kobori Y, Ban S, Okada H, Saito K (2020). SRY-Positive 46, XX Testicular Disorder of Sexual Development With Leydig Cell Tumor. Am J Mens Health.

[6] Xie LWY, Wang C, Xiang J, Wang H (2022). Genetic analysis and pathological features of one 46, XX testicular disorder of sex development cases with prostate germ cell tumor. Zhonghua Yi Xue Yi Chuan Xue Za Zhi.

[7] McElreavey K, Vilain E, Abbas N, Herskowitz I, Fellous M (1993). A regulatory cascade hypothesis for mammalian sex determination: SRY represses a negative regulator of male development. P Natl Acad Sci USA.

[8] Xia XY, Cui YX, Lu HY, Yang B, Wang GH, Pan LJ, Hou BS, Ge YF, Shao Y, Yao B (2007). Clinical, molecular and cytogenetic studies on 4 patients with 46, XX (SRY positive) male syndrome. Zhonghua Nan Ke Xue.

[9] Shi D, Zhang Y, Zhou Y, Mao Q, Li H (2020). Prenatal diagnosis of a fetus with 46, XX (SRY positive) male syndrome. Zhonghua Yi Xue Yi Chuan Xue Za Zhi.

[10] Margarit E, Coll MD, Oliva R, Gómez D, Soler A, Ballesta F (2000). SRY gene transferred to the long arm of the X chromosome in a Y-positive XX true hermaphrodite. Am J Med Genet.

[11] Baziz M, Hamouli-Said Z, Ratbi I, Habel M, Guaoua S, Sbiti A, Sefiani A (2016). Cytogenetic investigation in a group of ten infertile men with non-obstructive azoospermia: first Algerian 46. XX Syndrome Iran J Public Health.

[12] Abbas N, McElreavey K, Leconiat M, Vilain E, Jaubert F, Berger R, Nihoul-Fekete C, Rappaport R, Fellous M (1993). Familial case of 46, XX male and 46, XX true hermaphrodite associated with a paternal-derived SRY-bearing X chromosome. Cr Acad Sci III-Vie.

[13] Xing Y, Ji X, Xiao B, Jiang WT, Hu Q, Hu J, Cao Y, Tao J (2012). Molecular and cytogenetic characterization of six 46, XX males due to translocations between the short arms of X and Y chromosomes. Zhonghua Yi Xue Yi Chuan Xue Za Zhi.

[14] Terribile M, Stizzo M, Manfredi C, Quattrone C, Bottone F, Giordano DR, Bellastella G, Arcaniolo D, De Sio M (2019). 46, XX Testicular Disorder of Sex Development (DSD): A Case Report and Systematic Review. Medicina (Kaunas).

[15] Schneider DT, Schuster AE, Fritsch MK, Calaminus G, Göbel U, Harms D, Lauer S, Olson T, Perlman EJ (2002). Genetic analysis of mediastinal nonseminomatous germ cell tumors in children and adolescents. Gene Chromosome Canc.

[16] Hersmus R, Kalfa N, de Leeuw B, Stoop H, Oosterhuis JW, de Krijger R, Wolffenbuttel KP, Drop SLS, Veitia RA, Fellous M (2008). FOXL2 and SOX9 as parameters of female and male gonadal differentiation in patients with various forms of disorders of sex development (DSD). J Pathol.

[17] Peters JA, Beckjord EB, Banda Ryan DR, Carr AG, Vadaparampil ST, Loud JT, Korde L, Greene MH (2008). Testicular cancer and genetics knowledge among familial testicular cancer family members. J Genet Couns.

[18] Richardson R, Shoumacher R, Fer M, Hande K, Forbes J, Oldham R, Greco F (1982). The unrecognized extragonadal germ cell cancer syndrome. J Urol.

[19] Utz DC, Buscemi MF (1971). Extragonadal testicular tumors. J Urol.

[20] Oosterhuis JW, Looijenga LHJ (2019). Human germ cell tumours from a developmental perspective. Nat Rev Cancer.

